# Delayed duodenal/gastric fistula resulting in persistent perihepatic abscesses as a late complication of laparoscopic cholecystectomy

**DOI:** 10.1093/jscr/rjae071

**Published:** 2024-02-29

**Authors:** Phuong Vu, Meelod Daneshvar, Jayakrishna Chintanaboina, Amir Fathi

**Affiliations:** Department of Surgery, University of California San Francisco, Fresno, CA 93701, United States; Department of Surgery, University of California San Francisco, Fresno, CA 93701, United States; Department of Gastroenterology and Hepatology, University of California San Francisco, Fresno, CA 93702, United States; Department of Surgery, University of California San Francisco, Fresno, CA 93701, United States

**Keywords:** duodenal fistula, cholecystectomy, delayed complication, perforation, retained gallstones

## Abstract

Since the early 1990’s, laparoscopic cholecystectomy has become the gold standard for the treatment of symptomatic gallbladder disease. Although the incidence of postoperative complications is generally lower with this approach, gallbladder perforation represents a serious risk that is among the most common complications of laparoscopic cholecystectomy. The sequalae that can follow iatrogenic perforation have not been well documented and only a few case reports exist in the current literature. In this paper we discuss two case reports of delayed perihepatic abscesses following prior laparoscopic cholecystectomy, ultimately resulting in fistulous tracts. The course of the disease is discussed along with the diagnostic workup and eventual successful management of the aforementioned complications. Treating enteric fistulae requires a systematic approach and is carried out in phases. Enteric fistula formation following laparoscopic cholecystectomy is a rare complication of retained gallstones that can present months to years following the index operation. Significant care should be taken to avoid perforation and all efforts should be made to retrieve stones if spillage occurs.

## Introduction

Cholecystectomy is among the most common surgical procedures performed in the USA [1]. The laparoscopic approach, accounting for almost 90% of all cholecystectomies, is now the standard of care for the surgical treatment of symptomatic gallstone disease, as this approach is associated with a lower incidence of adverse events, morbidity, and mortality when compared with the open approach [2]. The reported complications of laparoscopic cholecystectomy range from 6 to 13% [3, 4]. The most common biliary complications include injury to the common bile duct and/or the right hepatic duct, and gallbladder perforation. Non-biliary complications include injury to nearby vascular structures, intestines, the diaphragm, and iatrogenic pneumothorax. Among the intraoperative complications, iatrogenic gallbladder perforation is one of the most common, often resulting in gallstone spillage [4]. Retained stones can lead to intra-abdominal abscesses, fistulae, and tumefactions of the abdominal wall. Only a few cases of such complications have been reported in the literature. Here, we report two cases of duodenal fistulae resulting in persistent perihepatic abscesses in patients with remote histories of laparoscopic cholecystectomy.

## Case report

### Case 1

**Figure 1:**
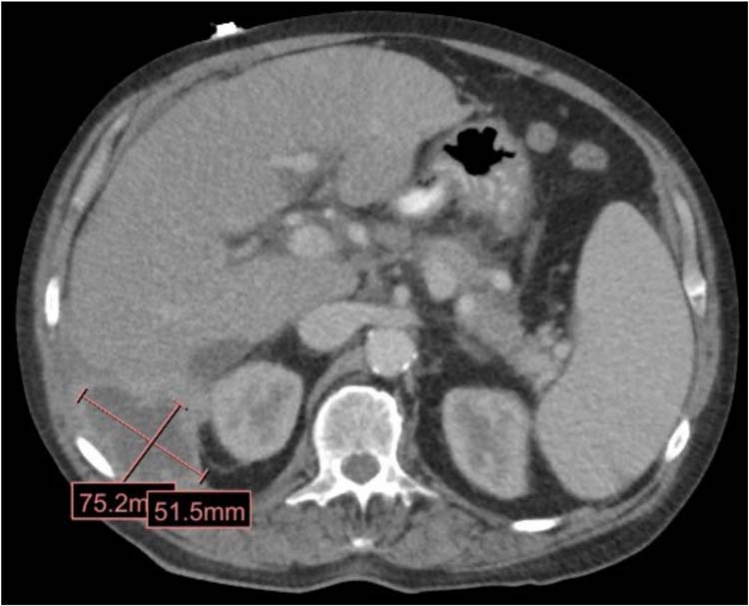
CT in the transverse plane demonstrating a perihepatic gallstone-containing collection of fluid measuring up to 7.5 × 5.1 cm.

**Figure 2:**
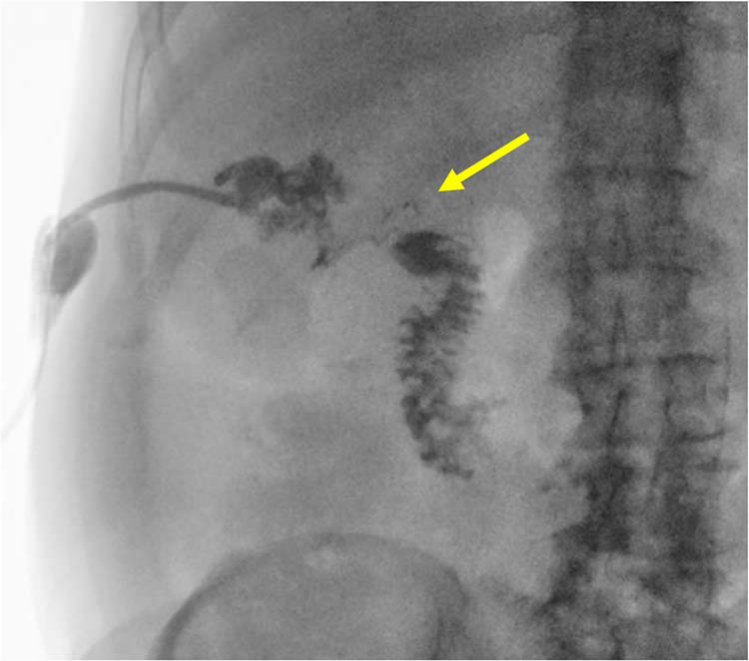
A radiographic fistulogram demonstrating the fistulous tract communicating with the second part of the duodenum.

**Figure 3:**
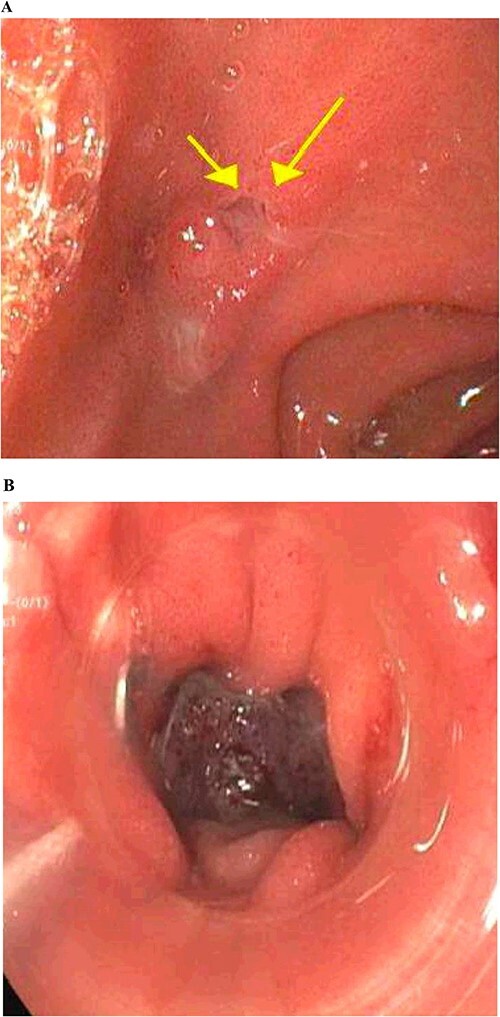
Gastrointestinal endoscopy. (A) Fistula defect found in the duodenal bulb. (B) Endoscopic closure using the over-the-scope clip system.

The patient is a 63-year-old male who underwent laparoscopic cholecystectomy with intraoperative cholangiogram for symptomatic gallstone disease at an outside hospital 2 years prior to our initial visit. His postoperative course was complicated by biloma and retained gallstones in the common bile duct, for which he underwent endoscopic biliary sphincterotomy 4 months after the initial surgery. His medical history was notable for alcoholic cirrhosis with a Model for End-Stage Liver Disease score of 14, duodenal ulcers, and gastric angiodysplasia. Symptoms included intermittent abdominal discomfort, nausea, and vomiting 1 year after the cholecystectomy. Computed tomography (CT) showed a perihepatic abscess measuring up to 7.5 × 5.1 cm, containing gallstones and punctate free-air near the right hepatic lobe (Fig. 1). He underwent image-guided percutaneous drainage of the hepatic abscess. A fistulogram via the abdominal drain demonstrated communication between the abscess cavity and the second segment of the duodenum, confirming the presence of a duodenal-perihepatic fistula, feeding the abscess cavity (Fig. 2). Interestingly, the oral contrast administered during a prior abdominal CT scan did not demonstrate this fistulous connection. The patient underwent endoscopic closure of the fistula using an over-the-scope clip device (Fig. 3). Repeat CT 2 months following the closure showed that the perihepatic abscess decreased in size with no evidence of a new abscess. On initial follow-up, the patient demonstrated clinical improvement in his overall health. Continued follow up revealed near complete resolution of the perihepatic abscesses and continued clinical improvement.

### Case 2

**Figure 4:**
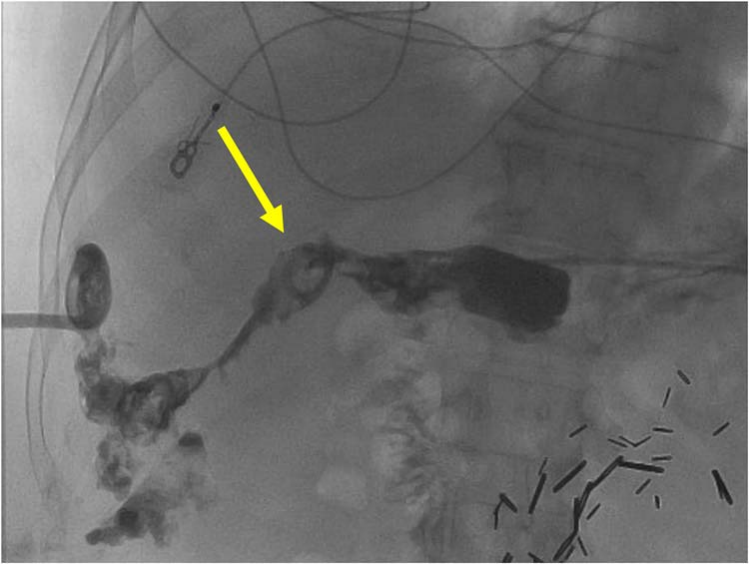
A radiographic fistulogram demonstrating the fistulous tract communicating with the proximal duodenum/gastric pylorus.

**Figure 5:**
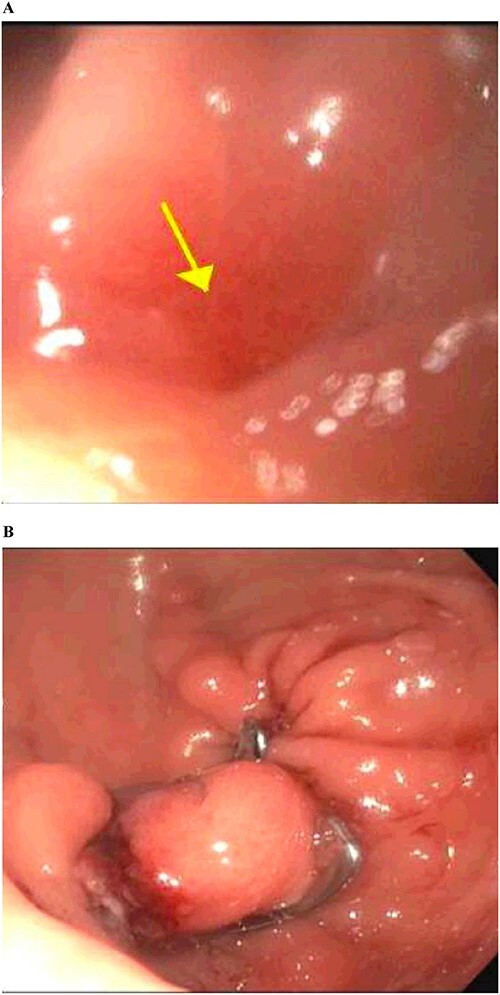
Gastrointestinal endoscopy. (A) Fistula defect found in the gastric antrum. (B) After endoscopic closure of the fistula using the over-the-scope clip system.

The patient is a 72-year-old male who underwent laparoscopic cholecystectomy for gangrenous cholecystitis at an outside hospital 23 months prior to our initial visit. Other pertinent medical history included ischemic stroke with resultant speech/vision deficits and left-sided hemiparesis, bladder cancer, testicular cancer, and colon cancer. His surgery was complicated by recurrent liver abscesses diagnosed 7 months postoperatively. He was treated with antibiotics and underwent multiple image-guided percutaneous drainages in the 9 months after his index operation. Unfortunately, many failed intravenous and oral courses of broad-spectrum antibiotics had resulted in treatment resistant *Clostridium difficile* infection, requiring stool transplant for successful eradication of his colonic infection. He presented to the emergency department 1 day after a drain placement procedure with complaints of right upper quadrant abdominal pain, nausea, and emesis. Similar to the first case presented in this report, the oral contrast administered during prior abdominal CT scan was unable to demonstrate this fistulous connection. Fistulogram performed through the abdominal drains demonstrated a fistulous connection between the abscesses and the gastric antrum (Fig. 4). Additionally, two small cutaneous fistulae in the right abdominal wall were identified, draining the overflow purulence from the perihepatic abscess cavities through the skin. He underwent endoscopic closure of the gastro-perihepatic fistula using the over-the-scope clip, followed by intraoperative abdominal wall fistulae washout 5 days later (Fig. 5). Both fistula cavities were packed with cotton packing strips, and the percutaneously placed drains were removed. He was discharged with oral antibiotics. Repeat CT scans at 1 and 3 months postoperatively demonstrated smaller perihepatic abscesses. Patient reported no symptoms of recurrence of abscesses or fistulae. The abdominal wall fistulae had decreased purulent drainage and continued to be managed with wet-to-dry packing.

## Discussion

Formation of fistula following laparoscopic cholecystectomy is a rare complication. Biliary fistula caused by iatrogenic injury during open or laparoscopic cholecystectomy occurs more often than enteric fistula, with an incidence of 0.3–0.6% of all cholecystectomies [5]. Duodenal fistula following laparoscopic cholecystectomy has been reported only once in the literature to the best of our knowledge [6]. Here we encountered two cases, a duodenal fistula and a gastric fistula, that presented to us 2 years after laparoscopic cholecystectomy.

Enteric fistula is often a sequela of surgical intervention, but can also occur spontaneously due to intra-abdominal inflammation or infection [7]. The etiology of enteric fistula following laparoscopic cholecystectomy is thought to be a complication of spilled and retained gallstones in the abdominal cavity following prior cholecystectomy. Zehetner and collegues [8] proposed that spilled gallstones propagate an inflammatory cascade that leads to abscess formation and even erosion of other abdominal organs. Pigmented stones, which are more prone to harbor bacteria, are more likely to cause abscesses and, subsequently, internal fistulization with adjacent organs [8].

Of the two cases we presented, one patient developed a duodenal fistula secondary to retained gallstone from the initial laparoscopic cholecystectomy. The other patient developed a gastric fistula for which an exact cause could not be established due to the limited past surgical records. We hypothesize that the cause could be lost stones or cautery injury. Spillage of gallstones from perforation of a gallbladder during dissection or extraction is a frequent complication of laparoscopic cholecystectomy. The estimated gallbladder perforation incidence ranges from 5 to 36% in patients with acute cholecystitis and occurs most often in the setting of gangrenous cholecystitis [9–11]. A review of 18 280 patients revealed that the incidences of gallbladder perforation and spillage were 18.3 and 7.3%, respectively [12]. Most of the spilled stones remain clinically asymptomatic. However, adverse events were reported in 0.04 to 19% of these cases [13]. A systemic review of 75 articles reporting the incidence of spilled gallstones from 2000 to 2016 showed that the most common complication is abscess formation, with intra-abdominal abscesses being the most common (36.5%) and fistula formation. These complications can manifest anywhere from 1 month to 15 years after index surgery [14]. Retained stones have been reported to create a fistula with the skin, colon, urinary bladder, and lungs [15]. Risk factors associated with complications after stone spillage are advanced age, male sex, acute cholecystitis, pigment stones, numerous stones (>15), stones with a diameter >1.5 cm, and perihepatic location of the retained stone(s) [16].

Patients usually present with abdominal symptoms such as abdominal pain, nausea, vomiting, poor appetite, diarrhea, and weight loss. These may be accompanied by fever and/or leukocytosis [7]. Imaging with contrast is typically used to confirm the presence and extent of the fistula. A CT scan is often done first as it can delineate not only the anatomy of the fistula, but also demonstrate any associated intra-abdominal fluid collection, abscess, or obstruction. If abdominal CT cannot elucidate the fistula anatomy, a gastrointestinal contrast study such as a small bowel follow-through or contrast enema can be performed. Alternatively, a fistulogram can be performed for enteric fistulas with a well-defined cutaneous opening by injecting a water-soluble contrast agent into the opening to define the fistulous tract.

Treating enteric fistulae requires a systematic approach and is carried out in phases. Initial acute management of enteric fistula focuses on control of infection, which requires surgery in some patients but can also, at times, be accomplished nonoperatively with antibiotics and catheter drainage. Once the source of infection is controlled, the treatment focuses on wound management, fluid/electrolyte replacement, and nutritional optimization. Most studies report that approximately one-third of enteric fistulae will close spontaneously within 5 to 6 weeks with conservative measures. However, endoscopic or surgical fistula repair is necessary for fistulae that fail to close spontaneously. In the cases we presented, the duodenal and gastric fistulae were successfully closed with endoscopic clipping of the intraluminal end of the fistula. The abdominal wall fistulae were surgically debrided and packed with wet-to-dry dressings.

## Conclusion

Enteric fistula is a rare complication of retained gallstones that can present many months following laparoscopic cholecystectomy. A systematic approach for diagnosis and treatment should be implemented in order to reduce morbidity and mortality.

## Lessons learned

Gallbladder perforation during laparoscopic cholecystectomy is among the most common intraoperative complications. Care should be taken to avoid gallbladder perforation during the procedure. All efforts should be made to retrieve the lost stones if spillage occurs. Furthermore, the incidence of spilled gallstones should be documented. Finally, clinicians need to suspect this in any patients with abdominal symptoms who have undergone a cholecystectomy in the past, even if the operation was performed remotely. Prompt diagnosis will ultimately help reduce morbidity and mortality rates.
